# IRE1α-XBP1 pathway promotes melanoma progression by regulating IL-6/STAT3 signaling

**DOI:** 10.1186/s12967-017-1147-2

**Published:** 2017-02-21

**Authors:** Cheng Chen, Xuejun Zhang

**Affiliations:** Department of Plastic Surgery, Zhongshan Hospital, Fudan University, Shanghai, 200032 China

**Keywords:** Melanoma, Proliferation, UPR, IRE1α, XBP1, IL-6, STAT3

## Abstract

**Background:**

The IRE1α-XBP1 pathway is the most conserved branch of the unfolded protein response pathways, which are activated during endoplasmic reticulum (ER) stress caused by the accumulation of unfolded/misfolded proteins in the ER lumen. The IRE1α-XBP1 pathway plays a critical role in various cancers. However, the role of this pathway in melanoma cell growth remains unclear.

**Methods:**

Sixty-one pairs of melanoma specimens and corresponding normal tissues from patients were stained with XBP1. Then, XBP1 splicing levels were detected in human tissues and cell lines at the mRNA level. IL-6 expression levels were determined in both melanocytes (HEMn-MP) and melanoma cells (Mel-RMu) overexpressing the spliced form of XBP1 (XBP1s). IL-6 expression was also examined in 4μ8C-treated HEMn-MP and Mel-RMu cells overexpressing IRE1α. Next, we analyzed potential XBP1s binding sites within the *IL*-*6* promoter and conducted ChIP experiments. IL-6/STAT3 signaling was detected by western blotting. Melanoma cell proliferation was examined by CCK8 and BrdU assays.

**Results:**

The mRNA and protein expression levels of XBP1s were significantly elevated in human melanoma tissues and cell lines compared with normal tissues or melanocytes, thus indicating the activation of the IRE1α-XBP1 branch in melanoma. Ectopic expression of IRE1α or XBP1s robustly enhanced IL-6 expression in HEMn-MP and Mel-RMu cells. Moreover, the inhibition of the RNase activity of IRE1α also abolished the effect of IRE1α in promoting IL-6 expression. Mechanistically, XBP1 binds the *IL*-*6* promoter and activates its expression. Furthermore, secreted IL-6 functions in an autocrine/paracrine manner, activates the intracellular JAK/STAT3 pathway and promotes the proliferation of melanoma cells.

**Conclusion:**

Our results reveal that the IRE1α-XBP1 pathway regulates Mel-RMu cell proliferation and progression by activating IL-6/STAT3 signaling.

## Background

Melanoma, one of the most malignant tumors, is increasing in incidence worldwide. However, there is still no curative treatment after the disease has spread beyond the primary site, owing to the proliferative ability of the cells [1]. Melanoma is infamous for its rapid proliferation rate [2], but until now, the exact mechanisms for the rapid proliferation of melanoma cells has remained unknown [3]. Because of poor vascularization and high proliferation rates, melanoma is subjected to many forms of stress. Unsurprisingly, these types of stress, including hypoxia, nutrient deprivation and altered pH, result in the accumulation of unfolded and/or misfolded proteins in the endoplasmic reticulum (ER) lumen and cause ER stress. This stress induces the activation of the unfolded protein response (UPR), which restores ER homeostasis, thus leading to cells producing more proteins for neoplastic growth, particularly secretory proteins. The UPR may assist in several aspects of tumor biology, including tumorigenesis, apoptotic evasion, metastasis, angiogenesis and chemotherapy resistance [3–6].

The UPR of mammalian cells is initiated by three ER transmembrane proteins: activating transcription factor 6 (ATF6), inositol-requiring enzyme 1 (IRE1), and double-stranded RNA-activated protein kinase-like ER kinase (PERK), which act as proximal sensors of ER stress. Under normal conditions, the luminal domains of these sensors are occupied by the ER chaperone glucose-regulated protein 78 (GRP78). Under ER stress, sequestration of GRP78 by unfolded proteins activates these sensors by inducing the phosphorylation and homodimerization of IRE1 and PERK along with the relocalization of ATF6 to the Golgi, where it is cleaved by Site 1 and 2 proteases, thus leading to its activation as a transcriptional factor [7–9]. After its activation, IRE1 catalyzes the non-conventional splicing of the mRNA encoding X-box-binding protein 1 (XBP1) by removing a 26 nt intron, thereby producing an active spliced form (XBP1s), thus initiating an essential UPR program [10].

A growing number of studies report that UPR is activated in various solid tumors; e.g., GRP78 expression is elevated in some cancers, including melanoma [11–13]. The spliced form of XBP1 is frequently expressed in melanoma cell lines and in fresh melanoma isolates [13]. Recently, it has been shown that UPR is be activated during early stages of melanoma initiation by the oncogenic form of HRAS (HRASG12V) [14]. The magnitude of the nascent protein production is higher in melanoma cell lines and results in the activation of UPR pathways, including the IRE1α-XBP1 branch [3, 4, 6].

The activation of signal transducer and activator of transcription 3 (STAT3) is usually transient in normal cells, but STAT3 has been reported to be present in a constitutively activated state and to promote tumorigenesis by enhancing cell proliferation, survival, and angiogenesis while suppressing the anticancer immune response in many different types of cancers, including colon cancer, melanoma and myeloma [15–17]. In some studies of lymphoid malignancies, interleukin-6 (IL-6) has been found to act in an autocrine/paracrine manner and to provide crucial survival signals by activating STAT3 signaling [17].

Here, we report that the IRE1α-XBP1 branch is activated and that the spliced form of XBP1 (XBP1s) is increased in human melanoma tissues. Compared with normal melanocytes, six melanoma cell lines showed higher XBP1 splicing and enhanced IL-6 expression. Ectopic expression of IRE1α or XBP1s gave rise to IL-6 expression, which in turn promoted Mel-RMu cell proliferation, whereas these effects were blocked by IL-6 antibodies. Further experiments revealed that XBP1s directly bound to the *IL*-*6* promoter and drove its expression. Our study reveals the crucial role of the IRE1α-XBP1 branch in promoting Mel-RMu cell proliferation by regulating IL-6/STAT3 signaling.

## Methods

### Patient characteristics

Clinical data, including age, sex, and the primary melanoma site, were collected retrospectively from patient records and their pathology reports. All patients were diagnosed with melanoma by the Department of Pathology, Zhongshan Hospital, Fudan University. In total, 61 patients were evaluated, and clinical and pathological data were analyzed for each patient. Of these patients, the youngest was 30 years old, and the oldest was 85 years old. The average age was 57.9 years, and the median age was 59 years. Thirty-six patients were male, and 25 patients were female. The primary sites of melanoma were grouped as head and neck, trunk and limbs, of which 75.41% were in the limbs (Table 1). All of the tumors were without regional or distant metastasis. The tissue sample collection was approved by the Ethics Committee of Zhongshan Hospital, Fudan University, and informed consent was obtained from all subjects. The tissue slides were prepared from biopsy paraffin blocks. The experiments were carried out under approved guidelines and complied with the 1975 Declaration of Helsinki.

**Table 1:** Clinical characteristics of patients with melanoma

Characteristics	No. (%)
Age	
≤40	9 (14.75)
40–60	24 (39.34)
≥60	28 (45.91)
Sex	
Male	36 (59.02)
Female	25 (40.98)
Primary sites of melanoma	
Head and face	3 (4.92)
Trunk	12 (19.67)
Limbs	46 (75.41)

### Immunohistochemical analysis

Immunohistochemistry was conducted by using anti-human antibodies against XBP1s (1:100, BioLegend, San Diego, CA, USA). The TMA slides stained with XBP1s were evaluated by light microscopy at 200× magnification by two investigators blinded to the clinicopathologic data of the patients. To access the expression intensity of XBP1s, the integrated absorbance in the area of a 1-mm-diameter cylinder was measured by using Image-Pro Plus version 6.0 (Media Cybernetics, Inc., Rockville, MD USA). The mean XBP1s density was calculated as the product of the integrated absorbance to total area. The tissue slides were prepared from biopsy paraffin blocks. The methods were carried out under the approved guidelines and complied with the 1975 Declaration of Helsinki.

### Cell culture

Melanocyte cell lines (HEMn-MP and HEMn-DP) and melanoma cell lines (Mel-RMu, MM200, Mel-CV, IgR3, A2058, and SkMel-28) were obtained from the Cell Bank of Shanghai, Chinese Academy of Sciences (Shanghai, China). All of the cell lines were cultured in high-glucose DMEM supplemented with 10% fetal bovine serum (Gibco, Thermo Fisher Scientific, Waltham, MA, USA).

### Quantitative real-time PCR

Real-time PCR analyses were performed as previously described [18, 19]. Briefly, total RNA of the cells was extracted using TRIzol reagent (Invitrogen, Waltham, MA, USA) according to the manufacturer’s protocol. Then, the RNA was reverse transcribed with an M-MLV first-strand cDNA synthesis kit (Invitrogen). Indicated mRNA levels were determined by qPCR using SYBR Premix Ex Taq (Roche, Basel, Switzerland), and human *GAPDH* was used as an internal control.

### Western blotting

Western blotting analysis was performed as previously described [20–22]. In brief, cells were harvested and lysed in RIPA lysis buffer. Then, proteins were separated by SDS-PAGE and transferred to polyvinylidene difluoride membranes. The membranes were washed in TBST, blocked in 10% milk, and then incubated with primary antibodies against human IRE1α (1:1000, Cell Signaling Technology, Boston, USA), XBP1s (1:500, BioLegend, San Diego, CA, USA), pSTAT3 (1:1000, Cell Signaling Technology), STAT3 (1:1000, Cell Signaling Technology) or GAPDH (1:5000, Abcam, Cambridge, UK) overnight at 4 °C, and this was followed by incubation with horseradish peroxidase-conjugated secondary antibodies. Proteins were detected with enhanced chemiluminescence assay (Thermo Fisher Scientific).

### CCK8 and BrdU assays

CCK8 assays were used to detect the effect of XBP1s on cell proliferation. Briefly, 1 × 10^3^ cells were seeded in 96-well culture plates, and these cells were then incubated with a CCK8 reagent for 2 h at 37 °C at the 24, 48, 72, 96 and 120 h time points. The staining intensity in the medium was measured by determining the absorbance at 450 nm.

BrdU assays were conducted by using a BrdU Cell Proliferation Assay Kit (#6813, Cell Signaling Technology, USA) according to the manufacturer’s instructions.

### Luciferase reporter assay

The pGL3 basic plasmid containing the promoter of the human *interleukin*-*6* gene, which corresponds to the region from −2000 to +100 nt on the putative transcription start site (denoted nucleotide +1), was constructed. The deletion of the ACGT core from the *IL*-*6* promoter was performed by using a PCR-based strategy. HEK293T cells were co-transfected with the designed plasmids. Luciferase activity was measured using a Dual-luciferase Assay Kit (Promega, Madison, WI, USA) according to the manufacturer’s instructions. *Renilla* luciferase activity was used as an internal control for normalization.

### Chromatin immunoprecipitation (ChIP)

ChIP assays were performed with an Agarose ChIP Kit (Pierce, Cat# 26156, Thermo Fisher Scientific), according to the manufacturer’s instructions. In brief, 293T cells were subjected to cross-linking with 1% formaldehyde, and glycine solution was then added to stop the cross-linking process. Nuclear extracts were prepared. Chromatin-XBP1s complexes were immunoprecipitated with anti-Flag (Sigma, Cat# F3165; diluted 1: 500, St. Louis, MO, USA) or anti-XBP1s (BioLegend, Cat# 647501; diluted 1:100) antibodies by incubation at 4 °C overnight, and this was followed by incubation with beads from the Agarose ChIP Kit (Pierce) or Protein G-Sepharose beads (GE Health, Chicago, IL, USA) at 4 °C for 1 h with gentle rocking. After the beads were washed 5 times with wash buffer, the complexes were eluted from the beads with elution buffer and subjected to PCR analysis.

### Statistical analysis

All experiments presented in this paper were repeated more than three times. The data are presented as the mean ± standard error of mean (s.e.m.). Statistical analysis (SPSS 18.0 software, SPSS Inc., Armonk, NY, USA) was performed with two-tailed independent Student’s t tests after a demonstration of homogeneity of variance with the F test or one-way ANOVA for more than two groups. Scheffe tests were used for post hoc analysis. The threshold for statistical significance was set at *P* < 0.05.

## Results

### Immunohistochemical staining to determine XBP1s expression in human melanoma tissues

To investigate the expression of XBP1s in human melanoma, immunohistochemical staining for XBP1s was conducted on a melanoma TMA containing 61 pairs of melanoma specimens and corresponding normal tissues. Compared with those in normal tissues, significantly enhanced protein levels of XBP1s were observed in melanoma tissues, thus indicating hyperactivation of the IRE1α-XBP1 pathway in human melanoma.

### The IRE1/XBP1 pathway regulates interleukin-6 expression in melanocytes and melanoma cell lines

**Fig. 1:**
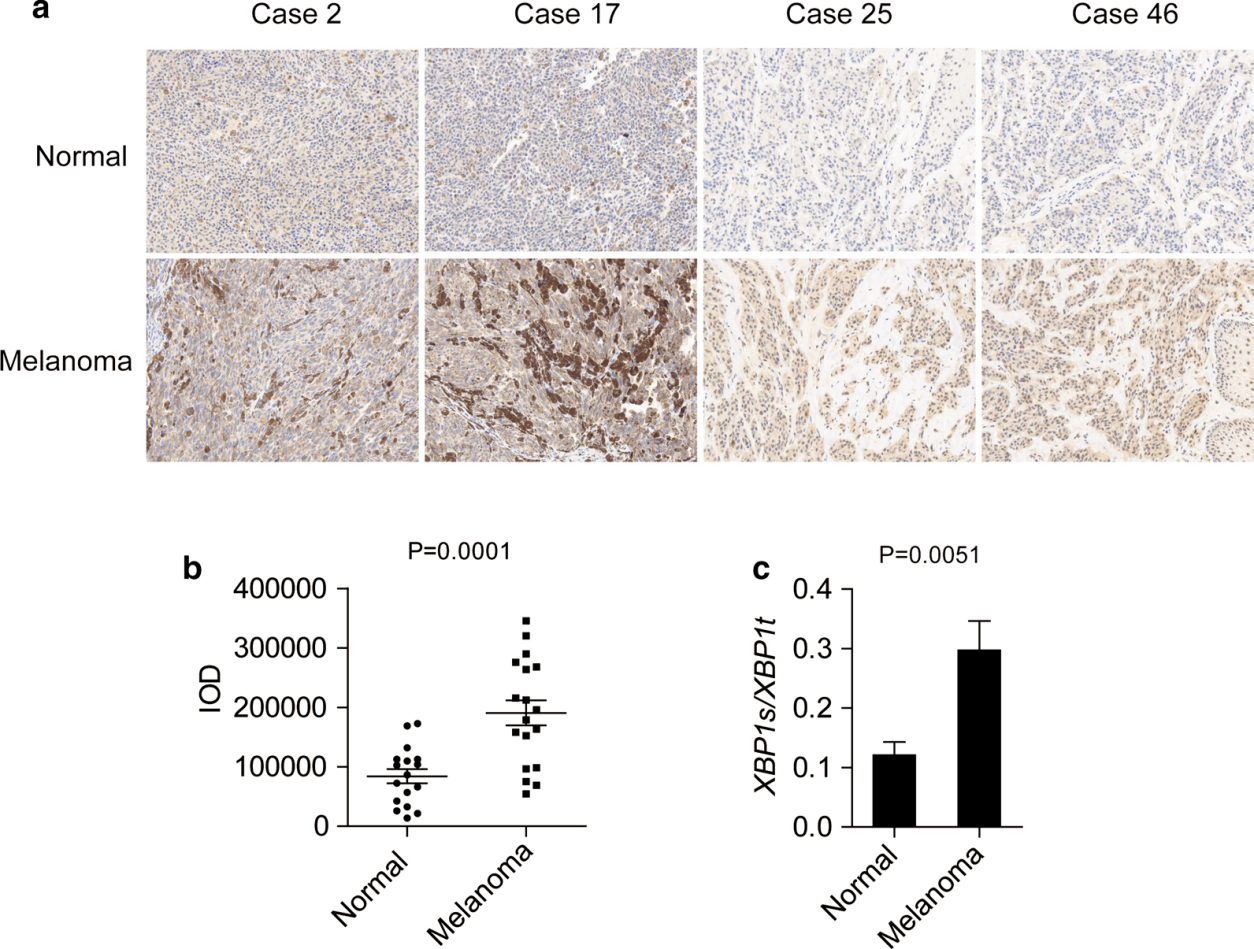
Expression of XBP1s in human melanoma samples. **a** Representative images and **b** integrated optical density (IOD) analysis of the immunohistochemical images of melanoma tissues and paired normal skin tissues from 61 melanoma patients. **c** XBP1 splicing levels were analyzed by real-time PCR in both melanoma tissues and normal skin tissues. The data are presented as the mean ± s.e.m., and P value was analyzed by Student’s t test and labeled

**Fig. 2:**
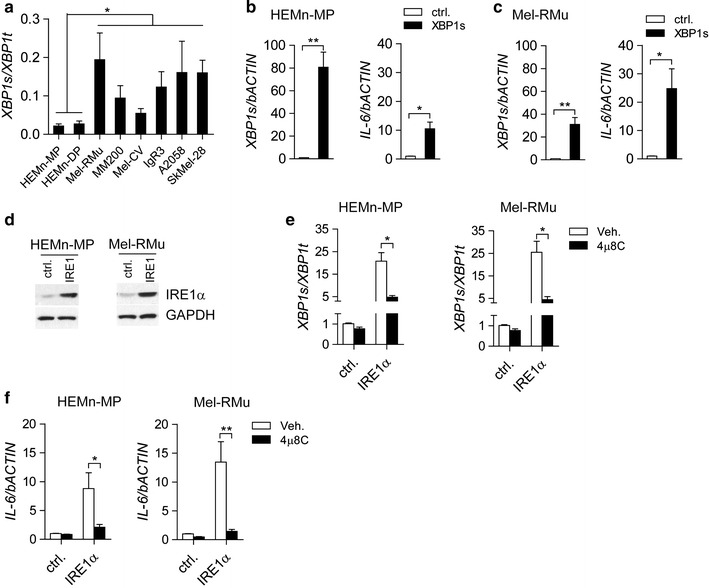
IRE1α-XBP1 pathway regulates interleukin-6 expression in melanocytes and melanoma cells. **a** XBP1 splicing levels in melanocytes (HEMn-MP and HEMn-DP) and melanoma cells (Mel-RMu, MM200, Mel-CV, IgR3, A2058 and SkMel-28). mRNA levels of *XBP1s* and *IL*-*6* as determined by real-time PCR in HEMn-MP (**b**) and Mel-RMu (**c**) cells transfected with plasmids pCMV-XBP1s (XBP1s) or pCMV (ctrl.). **d** The IRE1α protein levels were determined by immunoblotting in both HEMn-MP and Mel-RMu cells transfected with plasmids pCMV-IRE1α (IRE1) or pCMV (ctrl.). XBP1 splicing (**e**) and IL-6 (**f**) expression levels were determined by real-time PCR in IRE1α-overexpressing HEMn-MP and Mel-RMu cells with 4μ8C treatment. The results are from at least three independent experiments. The data are presented as the mean ± s.e.m. *P < *0.05*, **P < *0.01* by Student’s t test or two-way ANOVA

The results shown in Fig. 1 indicate that the IRE1α-XBP1 pathway is constitutively activated in human melanoma tissues. To investigate the splicing level of *XBP1* in melanoma cells, we analyzed mRNA levels of the spliced form of XBP1 (XBP1s) in a group of cell lines containing normal melanocytes (HEMn-MP and HEMn-DP) and melanoma cells (Mel-RMu, MM200, Mel-CV, IgR3, A2058 and SkMel-28). Compared with that in HEMn-MP and HEMn-DP cells, higher levels of XBP1u (unspliced XBP1) mRNA were spliced into XBP1s (spliced XBP1) and then translated into the active form of the protein in the melanoma cells, thus indicating constitutive activation of the IRE1α-XBP1 branch in melanoma cell lines (Fig. 2a).

In agreement with results from previous studies, the magnitude of nascent protein production was found to be higher in the melanoma cell lines than in the melanocytes, thus potentially leading to the activation of UPR pathways, including the IRE1α-XBP1 branch [3, 4, 6].

Next, we overexpressed XBP1s in melanocytes (HEMn-MP) and melanoma cells (Mel-RMu). As shown in Fig. 2b, c, IL-6 expression levels were dramatically increased in XBP1s—overexpressing HEMn-MP (Fig. 2b) and Mel-RMu (Fig. 2c) cells.

We then sought to determine whether IRE1α, the central mediator in the XBP1 splicing process, is involved in the regulation of IL-6 expression in melanoma. IRE1α was ectopically expressed in HEMn-MP and Mel-RMu cells (Fig. 2d). Owing to the excess accumulation of IRE1α, which leads to trans-autophosphorylation and activation of the RNase activity of the protein, XBP1 splicing levels were dramatically increased in the IRE1α-overexpressing HEMn-MP and Mel-RMu cells (Fig. 2e) but were blocked by the addition of 4μ8C, a specific inhibitor of the RNase activity of IRE1α (Fig. 2e). Moreover, with the increase in the level of the spliced form of XBP1, IL-6 expression was strongly enhanced in the IRE1α-overexpressing cells and was impaired after treatment with 4μ8C (Fig. 2f). Together, these results demonstrate that the upregulation of IRE1α and XBP1s activates the expression of IL-6 in HEMn-MP and Mel-RMu cells.

### XBP1s activates IL-6 expression by binding to the *IL*-*6* promoter in melanoma cells

**Fig. 3:**
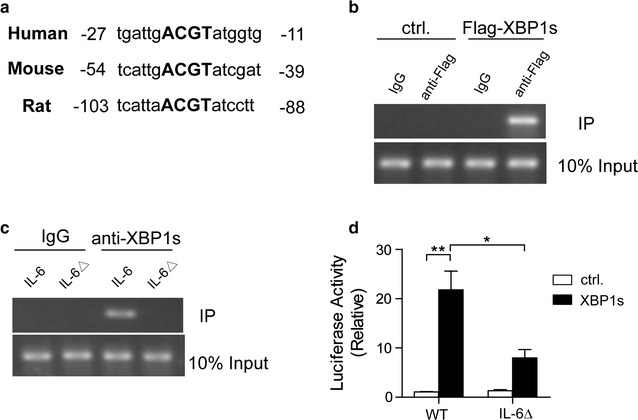
XBP1 activates IL-6 expression via binding to the *IL*-*6* promoter in melanoma cells. **a** Sequence alignment of the putative UPR element of the *Il*-*6* promoter from humans, rats and mice. The ACGT core is indicated in *bold*. **b** Mel-RMu cells were transfected with plasmids expressing Flag-tagged XBP1s or control (ctrl.). Chromatin immunoprecipitation (ChIP) was performed by using control IgG or anti-Flag antibody before PCR amplification of the indicated region of the *IL*-*6* promoter. **c** ChIP assays of extracts from 293T cells co-transfected with the empty vector or pCMV-XBP1s plasmid together with the *IL*-*6* or *IL*-*6* without the ACGT core (IL-6Δ) promoter constructs, using anti-XBP1s antibody. **d** Luciferase reporter assays were performed in 293T cells cotransfected with pCMV-XBP1s plasmid together with Luc constructs under the control of the wild-type (WT) human *IL*-*6* promoter or that with the ACGT core deleted (*IL*-*6*Δ). The results are from at least three independent experiments. The data are presented as the mean ± s.e.m. *P < *0.05,* **P < *0.01* by two-way ANOVA

To investigate whether XBP1s drives the transcription of IL-6 in melanoma cells, we analyzed the promoter sequences of human, rat and mouse IL-6 and identified a conserved putative UPR element containing the “ACGT” core sequence (Fig. 3a), a potential XBP1s binding site [23].

Next, ChIP experiments were conducted to address the exact mechanism(s). With an ectopic expression of XBP1s, antibodies specific to the flag-tagged XBP1s protein co-immunoprecipitated the *IL*-*6* promoter regions in Mel-RMu cells (Fig. 3b). Accordingly, the deletion of the “ACGT” core sequence in the *IL*-*6* promoter (*IL*-*6*Δ) led to loss in the ability of exogenous XBP1s to interact with the DNA containing the *IL*-*6* promoter in 293T cells (Fig. 3c). Furthermore, the results of luciferase reporter assays performed in 293T cells showed that ectopically expressed XBP1s robustly induced the transcriptional activity of the *IL*-*6* promoter; however, this effect was abolished in the *IL*-*6* promoter without the “ACGT” core sequence (Fig. 3d). These results demonstrate that XBP1s binds directly to the *IL*-*6* promoter and activates its transcription.

### The IRE1α-XBP1 branch promotes melanoma cell proliferation by regulating IL-6/STAT3 signaling

**Fig. 4:**
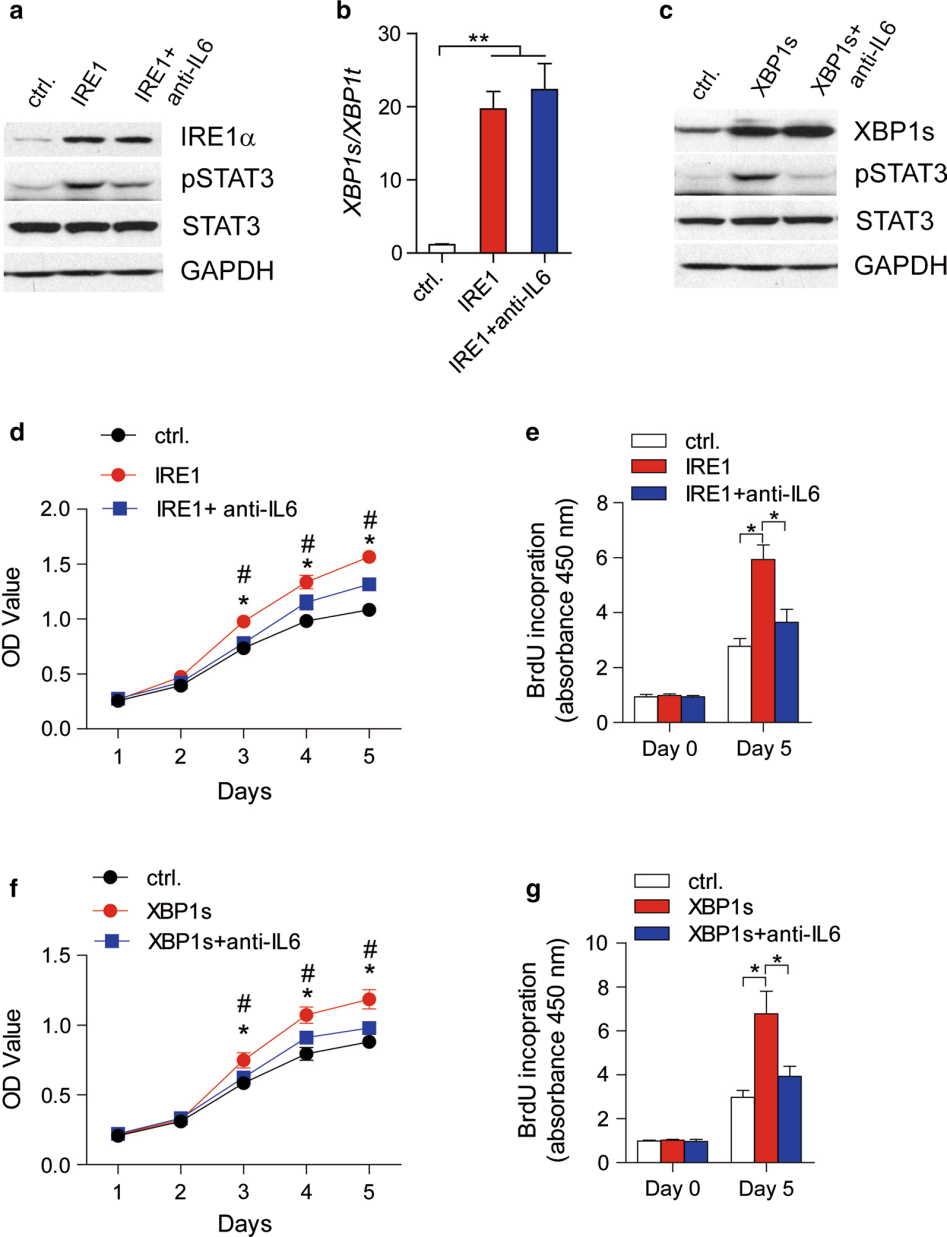
IRE1α-XBP1 pathway promotes melanoma cell proliferation by regulating IL-6/STAT3 signaling. **a** Immunoblotting analysis of the indicated proteins and **b** XBP1 splicing levels, as determined by real-time PCR in Mel-RMu cells transfected with plasmids pCMV-IRE1α (IRE1) or pCMV (ctrl.) and treated with anti-IL-6 antibodies (anti-IL-6). **c** Immunoblotting analysis of the indicated proteins in Mel-RMu cells transfected with plasmids pCMV-XBP1 (XBP1s) or pCMV (ctrl.) and treated with anti-IL-6 antibodies (anti-IL-6). **d**, **e** Cell proliferation was analyzed by CCK8 assay (**d**) and BrdU assay (**e**) in Mel-RMu cells transfected with plasmids pCMV-IRE1α (IRE1) or pCMV (ctrl.) and treated with anti-IL-6 antibodies (anti-IL-6). **f**, **g** Cell proliferation analyzed by CCK8 assay (**f**) and BrdU assay (**g**) in Mel-RMu cells transfected with plasmids pCMV-XBP1 (IRE1) or pCMV (ctrl.) and treated with anti-IL-6 antibodies (anti-IL-6). The results are from more than three independent experiments. The data are presented as the mean ± s.e.m. *P < *0.05,* **P < *0.01* by Student’s t test, one-way or two-way ANOVA relative to the ctrl. group; ^#^ *P* < *0.05* by Student’s t-test relative to IRE1 + anti-IL6 or XBP1s + anti-IL6 groups, as indicated

To investigate the effect of upregulated IL-6 induced by the activation of the IRE1α-XBP1 branch, we evaluated the phosphorylation levels of STAT3 in Mel-RMu cells with IRE1α or XBP1s overexpression. Strikingly, a robust increase in STAT3 phosphorylation was observed in cells with upregulated IRE1α (Fig. 4a) or XBP1s (Fig. 4c). To determine whether the increase in phosphorylated STAT3 was caused by extracellular IL-6 secreted by melanoma cells, we applied IL-6 antibodies to neutralize IL-6 in the medium. Interestingly, the addition of anti-IL-6 antibodies did not disturb the protein levels of IRE1α (Fig. 4a) and XBP1s (Fig. 4c) and did not affect IRE1α-mediated XBP1 splicing (Fig. 4b) but almost completely abolished the increase in STAT3 phosphorylation induced by the overexpression of IRE1α (Fig. 4a) or XBP1s (Fig. 4c) in Mel-RMu cells. These data suggested that the IRE1α-XBP1 pathway regulates the activation of STAT3 signaling by promoting IL-6 secretion by melanoma cells.

Many studies have revealed the critical role of IL-6/STAT3 signaling in regulating cell proliferation and tissue regeneration [15–17]. Hence, we sought to evaluate the role of the IRE1α-XBP1 branch in melanoma cell proliferation by performing CCK8 and BrdU assays in Mel-RMu cells. The results are shown in Fig. 4d–g. The proliferation rate of IRE1α-overexpressing cells was much higher than that of control cells, but proliferation was blocked by the addition of IL-6 antibodies (Fig. 4d, e). A similar phenomenon was observed in XBP1s-overexpressing melanoma cells (Fig. 4f, g). Together, our data revealed that the crucial role of the IRE1α-XBP1 branch in promoting Mel-RMu cell proliferation is exerted by regulating IL-6/STAT3 signaling.

## Discussion

UPR pathways, including the IRE1α-XBP1 branch, have been shown to have critical functions in the development of melanoma, but the exact mechanisms have been unclear. In our present work, we found that XBP1s levels were dramatically elevated in human melanoma tissues and melanoma cell lines compared with normal tissues or melanocytes. Ectopic expression of IRE1α and XBP1s in melanocytes and melanoma cells increased IL-6 levels and then activated intracellular STAT3 signaling, which was diminished by the addition of IL-6 antibodies. We also demonstrated that XBP1s bound to the *IL*-*6* promoter and activated *IL*-*6* transcription directly. Furthermore, increased expression of IRE1α and XBP1s promoted Mel-RMu cell proliferation, which was dependent on secreted extracellular IL-6.

XBP1s, the potent transcription activator and product of the RNase activity of IRE1α, mediates the expression of a large group of genes, thus relieving ER stress and reestablishing ER homeostasis [7–9, 24]. In addition to its critical role in ER stress, growing evidence has demonstrated that the IRE1α-XBP1 pathway controls the expression of many genes that are involved in the regulation of various biological processes, such as peroxisome proliferator-activated receptor alpha (PPARα) [18], protein disulfide isomerase (PDI) [25], fatty acid synthase (Fasn), and UDP-galactose-4-epimerase [26]. The IRE1α-XBP1 pathway also exerts non-transcriptional actions such as promoting the degradation of the forkhead box O1 (FoxO1) protein [27]. XBP1s was also found to activate *IL*-*6* transcription by binding to its promoter in murine macrophages involved in innate immunity [28]. Moreover, Toosi et al. have reported that vitiligo-induced activation of UPR pathways upregulates the expression of IL-6 and IL-8 in melanoma cells, but the molecular mechanism is not clearly defined [29]. Here, we demonstrated that XBP1s activated *IL*-*6* expression by binding to its promoter in human melanoma cells, thus indicating the conservation of XBP1 behavior in controlling IL-6 expression. Inhibition of the RNase activity of IRE1α by 4μ8C impaired IL-6 expression induced by the activation of the IRE1α-XBP1 pathway. Because IL-6 is considered to be a critical player in promoting cell proliferation [30] and progression and even a prognostic biomarker [30, 31] of melanoma, the RNase activity of IRE1α may be a promising therapeutic target.

Recently, Liu et al. have reported that IRE1α promotes hepatocyte proliferation and liver regeneration by regulating the STAT3 signaling pathway [32]. Although IRE1α has been implicated in cell proliferation in pancreatic islet cells [33] and certain cancer cell lines [34], it remains unclear whether the IRE1α-XBP1 branch is linked to melanoma cell growth. Our work is the first to demonstrate the critical role of IRE1α-XBP1s in promoting cell proliferation of melanoma and its detailed molecular mechanism.

Constitutive activation of STAT3 signaling has been observed in aggressive forms of cancer and is crucial in regulating tumor cell proliferation and survival in diverse cancer types [15, 35, 36]. Previous studies have revealed that STAT3 is constitutively activated in approximately 50–90% of melanomas [37, 38], but the exact details were unknown. Our results suggested that in melanoma cells, the constitutive activation of STAT3 is activated by secreted extracellular IL-6 working in an autocrine/paracrine manner, which can be abolished by adding IL-6 antibodies to the medium, thus neutralizing IL-6. Above all, IL-6 neutralization attenuated the enhanced proliferation of melanoma cells induced by the activation of the IRE1α-XBP1 branch, thus suggesting that anti-IL-6 antibody is a promising candidate for the clinical treatment of melanoma.

## Conclusion

In summary, our results reveal a novel role of the IRE1α-XBP1 branch of UPR pathways in regulating Mel-RMu cell proliferation and progression via controlling IL-6 expression and STAT3 signaling. However, in vivo studies are needed to clarify the high rate of melanoma cell proliferation caused by the constitutive activation of STAT3 signaling resulting from XBP1s-driven IL-6 expression. Our study provides a new promising therapeutic target for melanoma treatment and drug discovery.

## Abbreviations

ER: endoplasmic reticulum
UPR: unfolded protein response
IRE1: inositol-requiring enzyme 1
ATF6: activating transcription factor 6
PERK: double-stranded RNA-activated protein kinase-like ER kinase
GRP78: glucose-regulated protein 78
XBP1: X-box-binding protein 1
XBP1s: spliced form of XBP1
XBP1u: unspliced form of XBP1
XBP1t: total of XBP1s and XBP1u
IL-6: interleukin-6
STAT3: signal transducer and activator of transcription 3
PPARα: peroxisome proliferator-activated receptor alpha
PDI: protein disulfide isomerase
Fasn: fatty acid synthase
FoxO1: forkhead box O1 transcription factor
IOD: integrated optical density
